# Inhibition of FAK-Actin Pathway by Indoxyl Sulfate Suppresses Microvascular Remodeling in a Mouse Model of Chronic Kidney Disease

**DOI:** 10.3390/cells15211946

**Published:** 2026-10-26

**Authors:** Sean Michael Carr, Tharmarajan Ramprasath, Ming-Hui Zou

**Affiliations:** 1 Center for Molecular and Translational Medicine, Georgia State University, Atlanta, GA 30303, USA; sean.michael.carr@emory.edu (S.M.C.);; 2 Department of Endocrinology and Metabolism, Tianjin Medical University General Hospital, Tianjin 300052, China

**Keywords:** microvascular remodeling, chronic kidney disease, focal adhesion kinase, indoxyl sulfate

## Abstract

**Objective:** Chronic kidney disease (CKD) worsens outcomes of peripheral artery diseases (PADs), partly through impaired vascular regeneration and microvascular remodeling. CKD promotes the accumulation of uremic toxins, but their contributions to microvascular dysfunction remain poorly understood. This study investigated the effects of uremic toxin indoxyl sulfate (IS) on vascular smooth muscle cell (VSMC) function and microvascular remodeling in a mouse model of CKD. **Methods:** C57BL6/J mice were fed 0.25% adenine diet for 10 days to induce CKD. CKD mice underwent hindlimb ligation surgery. VSMC migration assays, microscopy, and Western blotting were utilized to elucidate the underlying mechanisms underlying the IS-induced impairment of vascular remodeling. **Results:** CKD mice exhibited significantly impaired blood flow restoration (44.7% relative blood flow) compared with controls (82.6% relative blood flow) accompanied by a reduced α-smooth muscle expression and decreased formation of smooth muscle-covered micro vessels in ischemic limbs. Wound healing and transwell migrations assays demonstrated IS significantly reduced VSMC motility. IS-exposure also disrupted actin cytoskeletal organization, impaired cell attachment, reduced cell size, and decreased focal adhesion formation. Mechanistically, IS reduced focal adhesion kinase (FAK) expression, impairing VSMC adhesion and migration. Reduced FAK signaling was associated with decreased N-WASP expression and suppression of the Arp2/3 complex, which regulates actin polymerization and cytoskeletal remodeling. These alterations suggest that IS disrupts the cellular processes required for VSMC recruitment and structural remodeling of the microvascular, potentially limiting vessel maturation and stabilization during ischemic recovery. **Conclusions:** IS impairs VSMC migration and cytoskeletal organization through the disruption of the FAK-N-WASP-Arp2/3 signaling axis, contributing to defective microvascular remodeling and impaired perfusion recovery in CKD-associated PAD. These findings identify uremic toxin-mediated cytoskeletal dysfunction as a potential therapeutic target for improving microvascular regeneration and vascular recovery in patients with CKD.

## 1. Introduction

Chronic kidney disease (CKD) is a serious medical condition that that imposes substantial medical and socioeconomic burdens, further exacerbated by its increasing prevalence [1]. Data from 1998 demonstrated, approximately 20 in every 1000 individuals over the age of 60 were affected by CKD; however, by 2012, that figure had more than doubled to 50 in every 1000 individuals in the United States [2,3,4].

CKD patients exhibit decreased renal filtration, resulting in the accumulation of uremic toxins and other solutes normally excreted by healthy kidneys. Uremic toxins have been implicated in the progression of multiple diseases and the exacerbation of preexisting conditions. Many of these compounds originate from amino acid degradation by gut microbiota. One extensively studied uremic toxin is indoxyl sulfate (IS), which is generated through bacterial metabolism of tryptophan [5]. Specifically, *E. coli* metabolize tryptophan to produce indole, which can function as a microbial signaling molecule. Following its release, indole crosses the intestinal barrier and enters the circulation, where it is transported to the liver and converted into IS through hydroxylation and is sulfonated. IS subsequently enters the circulation, binds strongly to albumin, and is transported to the kidneys for excretion. However, impaired renal function in CKD promotes IS accumulation, contributing to cardiovascular complications and increased mortality [6,7].

Cardiovascular disease is a leading cause of mortality in CKD patients, particularly during advanced stages of disease progression. Cardiovascular complications have been linked to the accumulation of uremic toxins, which contribute to or exacerbate dilated cardiomyopathy, coronary artery disease, atherosclerosis, and peripheral artery disease (PAD). Studies have demonstrated CKD patients are particularly susceptible to PAD which leads to tissue ischemia, extremity ulcerations, and infections [8,9]. Approximately 25.2% CKD patients have been reported to suffer from PAD, which impairs blood flow and wound healing, and increases the risk of lower extremity amputation [8]. PAD causes the narrowing or obstruction of peripheral arteries, reducing blood flow to distal tissues and increasing reliance on collateral circulation ana microvascular remodeling to restore perfusion [10]. CKD patients also experience vascular complications including bleeding or leakage at canulation sites, which interferes with hemodialysis [11]. Furthermore, CKD patients are at increased risk of adverse outcomes from abdominal aortic aneurysm repair and due in part to vascular dysfunction involving endothelial cells and vascular smooth muscle cells (VSMCs) [9]. Impaired vascular repair and inadequate blood flow restoration can further increase morbidity and mortality. Previous studies have demonstrated that CKD disrupts vascular structure and function, potentially contributing to impaired vascular regeneration, microvascular remodeling, and vessel maturation [12,13,14].

Vascular regeneration occurs through complementary processes: angiogenesis, collateral vessel formation, and microvascular maturation. Angiogenesis involves the formation of new capillary networks from preexisting vessels to restore microvascular perfusion, while vascular remodeling promotes structural adaptation of existing vessels to accommodate changes in blood flow and tissue demand. Together, these processes restore functional vascular networks and support tissue recovery following ischemic injury [14,15,16]. Vascular regeneration initially relies on endothelial cell activation, proliferation, and migration to form new capillaries that deliver oxygen and nutrients to damaged tissues. However, newly formed capillaries may remain structurally immature and insufficient to sustain effective tissue perfusion without subsequent stabilization and remodeling. Microvascular maturation requires the recruitment and integration of VSMCs to provide structural support, regulate vascular tone, and maintain vessel stability [17]. As developing micro vessels experience changes in blood flow, shear stress, and vascular pressure, they undergo structural remodeling to accommodate local hemodynamic demands. VSMCs contribute to the maturation and remodeling of small muscular vessels and arterioles by supporting vessel wall organization and adaptation to increase of blood flow [18]. The disruption of these processes can produce unstable and poorly functioning microvascular networks, limiting oxygen and nutrient delivery to ischemic tissues and impairing tissue recovery. Importantly, effective microvascular remodeling depends not only on cell availability but also intact cellular adhesion, cytoskeletal organization, and migration pathways required for VSMC recruitment and vessel stabilization. However, the molecular mechanisms underlying impaired microvascular remodeling a maturation in CKD remain poorly understood.

Cellular attachment and migration are regulated by multiple interconnected pathways involving focal adhesion complexes and cytoskeletal organization [19,20,21,22]. Focal adhesions are dynamic multiprotein complexes that mediate cell extracellular matrix interactions and transmit extracellular signals to intracellular pathways controlling cellular behavior. Responsiveness to these signals requires the coordination of canonical focal adhesion proteins including focal adhesion kinase (FAK), paxillin, Src, p130cas, actopaxillin, vinculin, talin, and many more [23,24,25,26]. Alterations in these complexes can impair cellular responses to extracellular stimuli, resulting in defective adhesion, migration, and d vascular repair. Focal adhesion signaling coordinates cell–matrix interactions with cytoskeletal remodeling by activating downstream effectors such as N-WASP. N-WASP (Neural Wiskott-Aldrich Syndrome Protein) is a key regulator of actin remodeling that activates the Arp2/3 complex to promote actin polymerization, membrane protrusions, and cell migration [26,27,28]. These processes are essential for VSMC motility and structural remodeling required for microvascular maturation. The disruption of FAK-dependent adhesion and N-WASP-Arp2/3-mediated actin polymerization may therefor impair VSMC recruitment, vessel wall organization, and microvascular stabilization. Defining how IS influences these pathways may provide important insight into the mechanisms underlying microvascular remodeling and impaired vascular regeneration in CKD patients.

In this study, we examined the role of IS in vascular regeneration and microvascular remodeling using a mouse model of CKD. Our findings demonstrate that CKD-associated IS exposure impairs VSMC migration and cytoskeletal organization through disruption of the FAK-N-WASP-Arp2/3-actin signaling axis. These alterations are associated with reduced smooth muscle coverage of small vessels and impaired perfusion recovery following ischemic injury, supporting a mechanism by which uremic toxins accumulation contributes to defective microvascular remodeling and vascular regeneration in CKD.

## 2. Materials and Methods

### 2.1. Animals

All animal experiments were approved by the Georgia State University Institutional Animal Care and Use Committee and conducted in accordance with the NIH Guide for the Care and Use of Laboratory Animals (Protocol Number: A21009, Date: 13 April 2021). Specific-pathogen-free mice were housed in the animal facility of Georgia State University under controlled conditions, including uniform temperature, humidity, and a 12 h light–dark cycle. The mice had unrestricted access to sterilized water and the designated diet. Mice were anesthetized with isoflurane (5% for induction, inhalation) until loss of reflex, followed by cervical dislocation for euthanasia and subsequent tissue collection.

#### 2.1.1. Establishment of the CKD Model and Experimental Design

To examine the effect of CKD has on vascular regeneration and microvascular maturation a CKD model (adenine diet) was utilized [13]. Mice were randomly assigned to following groups: Nine mice (CKD group) were fed a 0.25% adenine diet for 10 days, followed by 4 days of recovery on a chow diet prior to surgery, to allow stabilization of kidney injury and clearance of residual adenine [29,30]. A limitation of this study is that the 10-day adenine diet represents a relatively acute model of renal dysfunction and may not fully recapitulate the progressive systemic changes characteristic of human CKD. Control mice (n = 8, control group) were maintained on a chow diet for the corresponding 14 days period prior to surgery. Hindlimb ligation was performed on the femoral artery and 3 branching arteries into the hindlimb as previously described [13]. Both groups were maintained on chow diet after surgery until mice sacrifice. Mice were examined for adverse effects from adenine diet and surgical procedures, and appropriate steps, including administration of pain medication, and supplementary water and food gel, were taken in the event of adverse outcomes. Blood flow restoration was examined at day 0 (pre-surgery) and on days 1, 3, 7, 14, 21, and 28 post-surgery using laser doppler.

#### 2.1.2. Ultrasound Analysis

To determine the effect of CKD on the cardiovascular system ultrasound analysis was employed. Analysis was conducted on day 28 immediately prior to euthanasia. Cardiac and vascular ultrasound analysis was performed using a Vevo 3100 High-Resolution Micro-Ultrasound System (FUJIFILM Visual Sonics Inc., Toronto, ON, Canada). For cardiac ultrasound, M-mode tracing was used to measure left ventricular wall thickness, left ventricular end-systolic diameter, and left ventricular end-diastolic diameter. Ejection fraction and fractional shortening were calculated from left ventricular dimensions in the 2D short-axis view. The vascular ultrasound was performed as previously described [31]. Systolic diameter and diastolic diameter were obtained from M-mode images and then circumferential cyclic strain was calculated as (systolic diameter − diastolic diameter)/diastolic diameter. The aortic pulse wave velocity (PWV) was calculated as the distance between 2 measurement points (L) divided by the time shift of the waveforms at the 2 points (i.e., PWV = L/(Δt_1_ − Δt_2_)). All examinations were analyzed using Vevo Lab 2.1.0 software (FUJIFILM Visual Sonics Inc., Toronto, ON, Canada).

#### 2.1.3. Terminal Procedures

After ultrasound, mice were euthanized, and tissues were collected for Western blot and immunofluorescence examination. Tissue was either stored in formalin fixative solution or −80 °C. Serum was collected and analyzed for creatinine (Arbor Assays, KB02-H1, Ann Arbor, MI, USA) and urea nitrogen (Arbor assays, K024-H1, Ann Arbor, MI, USA). Serum was collected from 5 CD mice and 6 AD mice this is due to collection issues. Animal experiments were done in accordance with Georgia State University Institutional Animal Care and Use Committee approved animal protocols (13 April 2021, A21009), ethical considerations, and under the supervision of veterinary personnel.

##### High-Performance Liquid Chromatography Determination of IS

Measurements of IS levels are determined to further confirm kidney dysfunction and demonstrate uremic toxin buildup. Sample preparation for serum was done by mixing 50 μL of sample by vortexing in a 1.5 mL Eppendorf tube with 1/10 volume of ice-cold 2.4 mol/L perchloric acid. The cloudy suspension was chilled on ice for 15 min and then centrifuged at 8000× *g* for 3 min. The clear protein-free supernatant was analyzed directly. IS content was estimated along with standards ranging from 0.250–25 μmol/L IS (I3875 Millipore Sigma, Rockville, MD, USA), using a C18 column (Symmetry C18 Column, 100 Å, 5 µm, 4.6 mm × 250 mm; Waters, Milford, MA, USA). The mobile phase was prepared using the acetonitrile/water (ACN/H2O: 66/34) and the injected sample volume was 20 μL. The flow rate was 1 mL/min at 38 °C. The column effluent was monitored at 278 nm by a UV detector.

##### Immunocytochemistry

Examination of damaged vasculature in the ligated gastrocnemius muscle occurred via immunofluorescent staining for von Willebrand factor (vWF) (endothelial cells) and alpha smooth muscle actin (α-SMA) (VSMCs) 28 d post-surgery [14]. Tissues were fixed in 10% ice-cold formalin overnight, then embedded in paraffin. Briefly, slides were deparaffinized and dehydrated for 30 min at room temperature. Sections were incubated for 30 min in diluted normal blocking serum, and primary antibodies were added to slides after excess serum had been removed. Respective antibodies against vWF and α-SMA (Santa Cruz Biotechnology, Dallas, TX, USA) were diluted according to the manufacturer’s instructions.

### 2.2. In Vitro Studies/Mechanistic Studies in VSMCs

#### 2.2.1. Cell Culture

Uremic toxins, specifically IS, which accumulate during CKD progression, are known to has adverse effects on vascular cells that contribute to cardiovascular disease [15]. VSMCs were examined due to observed loss in ligated muscle tissue. Multiple IS concentrations, from moderate to severe CKD levels, were assessed, and it was determined that 250 μmol/L was the optimal concentration for in vitro experiments. VSMCs were purchased from American Type Culture Collection and maintained in either Smooth muscle growth media (SmGM, Lonza, Walkersville, MD, USA) or Medium 231 (Gibeco, Bilings, MT, Life Technologies Corporation, Grand Island, NY, USA) with smooth muscle cell supplements and 10% fetal bovine serum (FBS) during growth phase. At 24 h prior to start of experiment, VSMCs were placed into media containing 2% FBS to synchronize cell cycle of all cells towards a quiescent state. All cells were maintained at 37 °C in 5% CO_2_ and 21% O_2_ normoxic or 1% O_2_ hypoxic conditions.

#### 2.2.2. Reagents and Cell Treatments

For experimentation, cells were divided into various treatment groups including IS stimulation, pathway inhibitor treatments, and corresponding vehicle control groups. For stimulation experiments, cells were treated with IS (Millipore Sigma, I3875, Millipore Sigma, Rockville, MD, USA) for 2 days in medium containing 2% serum. We utilized 250 μM IS as a moderate, pathophysiologic ally relevant concentration to model CKD-associated uremic conditions, as higher IS concentrations are generally associated with greater uremic burden and more advanced CKD. To inhibit the lysosomal activity, cells were treated with bafilomycin A1 (BafA, Millipore Sigma, Rockville, MD, USA) and Chloroquine (CQ, Millipore Sigma, Rockville, MD, USA) for 8 h. To block proteasome degradation, MG132 (Invitrogen, Waltham, MA, USA) was used for 8 h. Control cells were treated with vehicle only (phosphate-buffered saline, Millipore Sigma, Rockville, MD, USA).

#### 2.2.3. Wound Healing and Transwell Migration Assay

To understand the effect that IS on the migratory ability of VSMCs, wound healing and transwell assays were employed. Wound healing assay was performed in VSMCs when they were confluent under 2% serum containing media. Wound was applied via a 200 μL pipette tip and migration proceeded for 12 h. Images were taken before and after migration. Area within the wound was calculated and analyzed using ImageJ (Version 1.51) (National Institutes of Health, Bethesda, MD, USA) and analyzed. Transwell migration assay was performed with 40,000 cells per 200 μL and migration proceeded for 6 h under 2% serum containing media. Cells were fixed in methanol-crystal violet solution and imaged.

#### 2.2.4. G-Actin/F-Actin Isolation

IS exposure impairs VSMC motility, likely by affecting the actin cytoskeleton. Given its key role in cell movement, we next examined how IS alters the actin cytoskeleton. Actin was isolated utilizing G-Actin/F-Actin in vivo Assay Biochem Kit (Cytoskeleton Inc., BK037, Denver, CO, USA). Control and 250 μmol/L IS pre-exposed VSMCs were utilized. Actin isolation was performed in accordance with kit protocol. Samples were run on western blot analysis to demonstrate the changes in actin structure.

#### 2.2.5. Cell Attachment and Size Assay

Since IS alters the actin cytoskeleton, we next examined whether it also affects VSMC morphology. VSMCs typically prefer binding to laminin, but this preference can change under disease conditions. To assess changes in cell attachment, size, and substrate preference, we used standard extracellular matrix (ECM)- or laminin-coated plates. ECM laminin (20 μg/mL), fibronectin (10 μg/mL), and liquid gelatin were diluted in PBS and incubated on plates for 1 h. Plates were blocked with 5% BSA for 1 h to prevent nonspecific binding. Control or 250 μmol/L pre-exposed VSMCs were plated on ECM for 6 h in 2% FBS media and then fixed with 4% paraformaldehyde. Stained with Phalloidin (1:1000) for 20 min and mounted with DAPI gold-mounting media (Thermo Fisher Scientific (Invitrogen), Waltham, MA, USA). Size was determined as previously described.

#### 2.2.6. Western Blots

To further understand how IS exposure affects cellular structure and attachment, we examined focal adhesions—critical sites for cell adhesion and extracellular signal transduction. Control and IS exposed VSMCs were examined for WB experiments. Specifically, we evaluated the expression of key focal adhesion proteins, FAK and paxillin. IS exposure led to a loss of FAK, disrupting downstream signaling pathways essential for actin fiber formation. We next investigated the downstream effector of FAK, Neuronal Wiskott-Aldrich Syndrome Protein (N-WASP), which is involved in actin polymerization via activation of actin-related complexes. Our initial step was to determine whether IS exposure alters N-WASP expression and, by extension, its activation. As previously described, Western blots were done, and blots were probed with specific antibodies. Primary antibodies used as follows: β-actin and GAPDH anti-mouse monoclonal antibody (Santa Cruz Biotechnology, Dallas, TX, USA) were used as internal controls; anti-rabbit polyclonal FAK, FAK (Y397), paxillin, paxillin (Y118), Hif1α, Arp2, and Arp3 (Cell Signaling, Danvers, MA, USA); anti-rabbit polyclonal N-WASP (Millipore Sigma, Rockville, MD, USA) were experimental proteins analyzed. After background subtraction from the calculated area, band intensities were measured and quantified by densitometry (GS-700 Imaging Densitometer; Bio-Rad (Hercules, CA, USA). Quantification of Western blot was based on the ratio of the target protein to GAPDH or β-actin housekeeping protein.

#### 2.2.7. siRNA Transfection

Given that IS-exposed VSMCs show reduced motility and loss of focal adhesion proteins, we next asked whether this loss directly impairs migration. To test this, we silenced FAK or paxillin in VSMCs and assessed their migration using a transwell assay. VSMCs were routinely passaged 24 h before transfection and transfected at approximately 70% confluency. Cells were transfected with either siRNA for FAK, Paxillin, or N-WASP (Santa Cruz Biotechnology, Dallas, TX, USA) for 6 h in Opti-MEM (Gibco, Billings, MT, USA) using the RNAImax (Invitrogen, Waltham, MA, USA), then incubated for 24 h in 2% FBS media, before incubation under normoxic or hypoxic conditions (1% O_2_; 12 h) or further manipulation.

#### 2.2.8. Light and Confocal Microscopy

Cells were plated on glass coverslips and fixed in 4% paraformaldehyde. Cells were permeabilized with either 0.1% Triton X-100 and blocked with 0.1% BSA before antibody addition. Indirect immunofluorescence experiments were performed using the following primary antibodies: anti-rabbit FAK (1:200; Cell Signaling, Danvers, MA, USA), anti-mouse N-WASP (1:50; Santa Cruz, Dallas, TX, USA), anti-mouse paxillin (1:50; Santa Cruz, Dallas, TX, USA), anti-rabbit N-WASP (1:50; Millipore Sigma, Rockville, MD, USA), and anti-mouse Arp2 (1:50; Santa Cruz, Dallas, TX, USA). Secondary antibodies consisted of Alexa Fluor 488, 555, and 647 (donkey anti-goat, -rabbit, or -mouse, respectively) used at 1:400 dilution.

#### 2.2.9. Plasmid Overexpression

We employed overexpression of FAK in VSMCs to further demonstrate the effect on motility. FAK overexpression plasmid (Origene, Rockville, MD 20850, USA, RC219839) and was transfected at a concentration of 500 ng/mL into VSMCs using optiMEM medium and Lipofectamine 2000 transfection kit (Thermo Fisher, Waltham, MA, USA) for 24 h prior to experimentation.

#### 2.2.10. Statistics

Data are expressed as mean ± SEM or mean ± SD as indicated in the figure legends. Statistical comparisons were performed using unpaired 2-tailed Student’s *t*-test, 1-way ANOVA, or 2-way ANOVA with Bonferroni multiple-comparison test. A *p* value less than 0.05 was considered significant (GraphPad, Boston, MA, USA).

## 3. Results

### 3.1. Elevated Levels of IS in Adenine Diet Model of CKD

**Figure 1:**
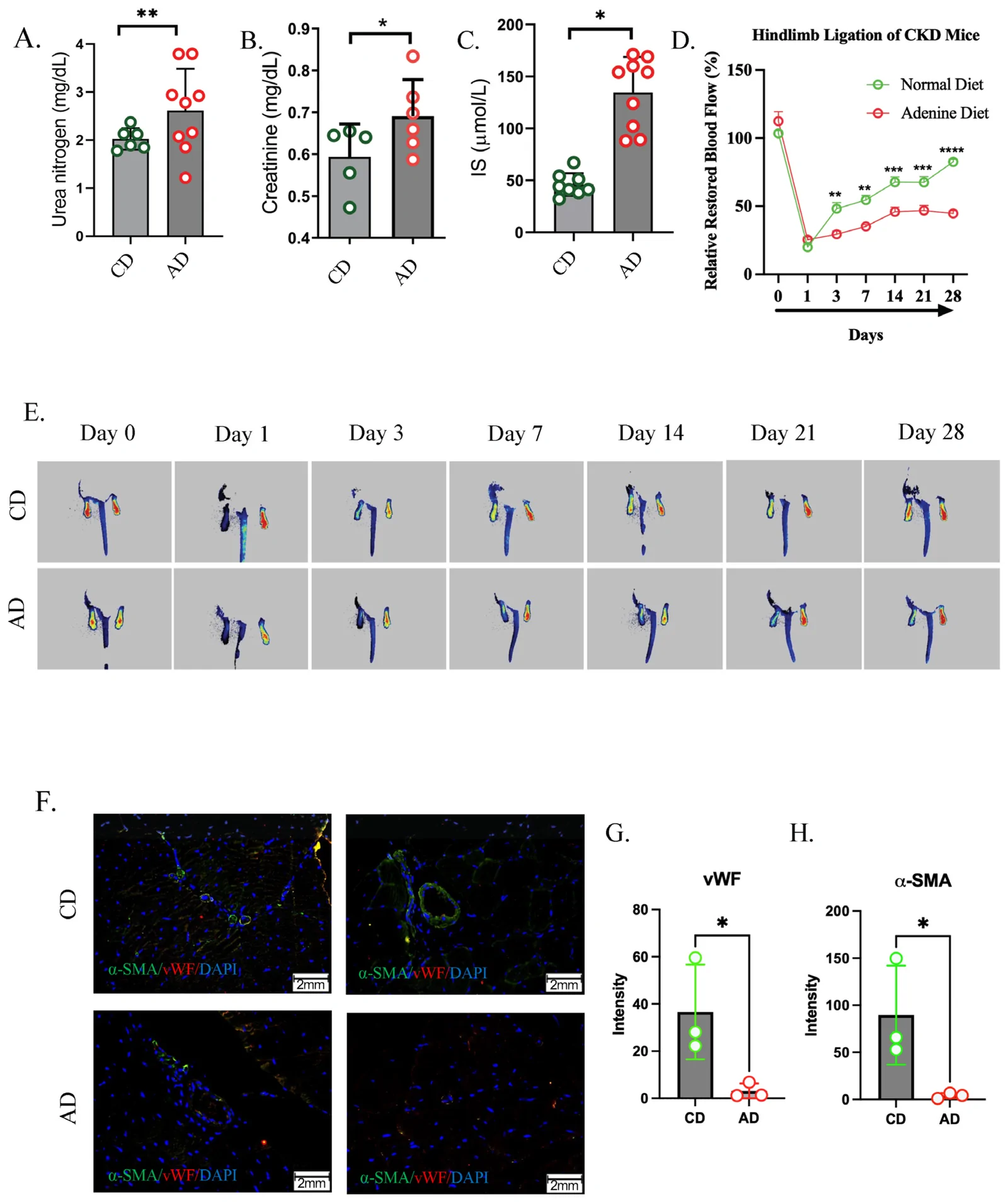
Hindlimb ligation and kidney dysfunction in WT mice. (**A**) Serum nitrogen levels are significantly elevated in AD mice (CD, n = 8; AD, n = 9). (**B**) Serum creatinine levels (CD, n = 8; AD, n = 9). (**C**) Serum indoxyl sulfate levels measured after CKD induction to demonstrate increased uremic toxin buildup (CD, n = 8; AD, n = 9). (**D**) Relative percentage of blood flow restoration after hindlimb ligation. AD group (AD, adenine diet) mice exhibited approximately 44.7% blood flow restoration compared to control (CD) mice 82.6% restoration of blood flow (CD, n = 9; AD, n = 8). 2-way ANOVA analysis was performed to demonstrate that under uremic conditions there is reduced vascular regeneration. (**E**) Heat map for blood flow restoration 28 days post-surgery. (**F**) Ligated gastrocnemius muscle was stained 28 days post-surgery for capillaries vWF (Red, endothelial cells), ⍺-SMA (Green, VSMCs) (n = 3). (**G**) Quantification of vWF from CD and AD ligated muscle. There is a decrease in intensity of vWF staining (n = 3). (**H**) Quantification of ⍺-SMA. There is a decreased staining for ⍺-SMA in ligated tissue (n = 3). * *p* < 0.05, ** *p* < 0.005, *** *p* < 0.001, **** *p* < 0.0001.

CKD induction was confirmed via significantly elevated levels of serum nitrogen and creatinine levels in the CKD group compared to control group (Figure 1A,B). The CKD group exhibited significantly increased levels of uremic toxin IS in serum (Figure 1C). The laser doppler analysis of CKD mice exhibited impaired blood flow restoration (44.7% restoration) compared to controls (82.6% restoration) after 28 d of recovery in the ligated hindlimb (Figure 1D,E) [13,28,29]. Cardiovascular function was examined due to previously reported complications associated with CKD. Mice of the CKD group presented with cardiovascular complications including a higher PWV and decreased fractional shortening and ejection fraction, but no change in strain (Supplementary Figure S1A–D).

### 3.2. Impaired Microvascular Remodeling in CKD

CKD mice exhibited impaired microvascular remodeling in the gastrocnemius muscles as evidenced by the reduced α-SMA expression in vWF-positive vessels (Figure 1F–H) [32]. These vessels exhibited reduced VSMC coverage compared to controls.

### 3.3. IS Exposure Impairs VSMC Motility

**Figure 2:**
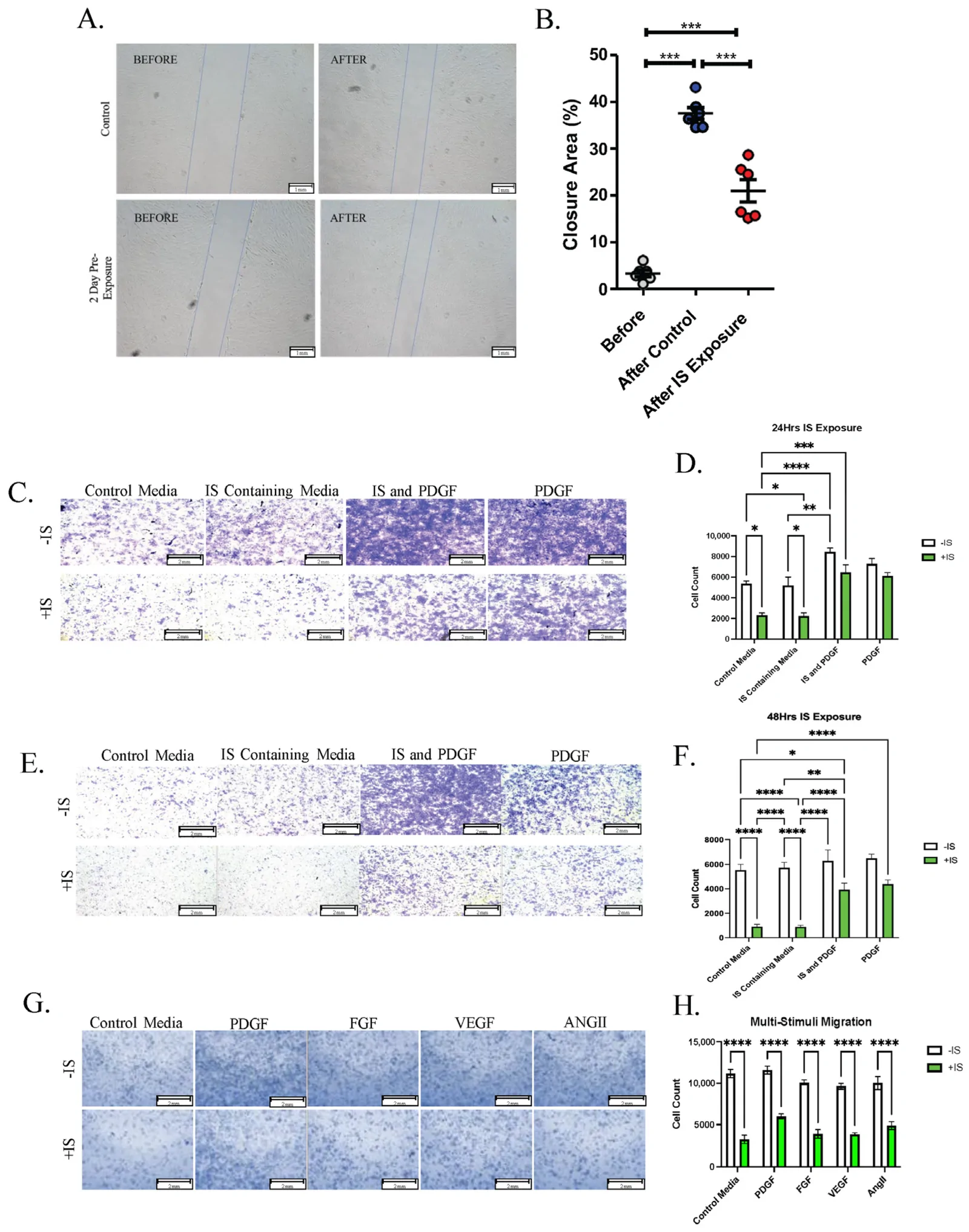
Indoxyl sulfate effect on VSMC wound healing and migration. (**A**,**B**) VSMCs exposed to indoxyl sulfate had decreased wound healing capacity and resulted in wound closure of approximately 20% compared to approximately 40% closure after 12 h (n = 6). (**C**) Transwell migration assay was utilized for VSMCs exposed to IS for 24 h. There was a signification decrease in VSMC migration. PDGF (10 ng/mL) was utilized as a positive control. PDGF stimulation did induce migration however IS-treated cells had significantly less migration than untreated cells (n = 3). (**D**) Quantification of 24 h transwell migration (**E**) 48 h of IS exposure was utilized. Transwell migration yielded similar results to the 24 h exposure. Migration was inhibited (n = 3). (**F**) Quantification of 48 h transwell migration. (**G**) Various other migration stimulators were utilized PDGF, FGF (1 ng/mL), VEGF (10 ng/mL), and Angiotensis II (ANGII, 5 µM). This demonstrated that chemotactic agents were unable to rescue migratory ability (n = 3). (**H**) Quantification of multi-stimuli transwell migration * *p* < 0.05, ** *p* < 0.005, *** *p* < 0.0005, **** *p* < 0.0001.

An examination of the migratory efficiency in IS-exposed VSMCs demonstrated a closure area of approximately 20%, while control samples exhibited an increased wound closure area of approximately 35% (Figure 2A,B) [33]. Next, a transwell migration assay was employed to further examine the IS-exposed VSMC migratory ability. IS-exposed VSMCs were applied to the transwell assay and, after 6 h migration, there was a marked reduction in migratory ability compared to the control. PDGF-ββ, a VSMC migration activator, only partially rescued the IS-exposed VSMC motility, lending evidence of IS-exposure (Figure 2C–F) [34]. It should be noted that the media did not contain PDGF-ββ and cells were kept in media containing 2% FBS for the duration of experiment. The utilization of migration activators FGF, VEGF, and ANGII was incapable of reversing IS impaired VSMC motility (Figure 2G,H). This data demonstrated that IS impaired motility can be ameliorated but not completely reversed. An examination of endothelial cell motility was undertaken due to their role in capillary formation and previous data that has demonstrated IS-exposure impaired migratory ability.

To ensure that our observations are related to decreased migration and not cell death, cell viability was examined. Cell viability decreases significantly until 4 days after exposure to IS but has a minimal effect after 2 days of exposure (Supplementary Figure S2A). IS-exposure does induce inflammation in VSMCs due to the expression of IL-6 (Supplementary Figure S2B). Our data now lends compelling evidence that IS-exposure directly inhibits motility and IS exposure significantly reduces cellular motility, providing direct evidence that IS contributes to the loss of migratory capacity in VSMCs.

### 3.4. IS Alters VSMC Actin Cytoskeleton and Attachment

**Figure 3:**
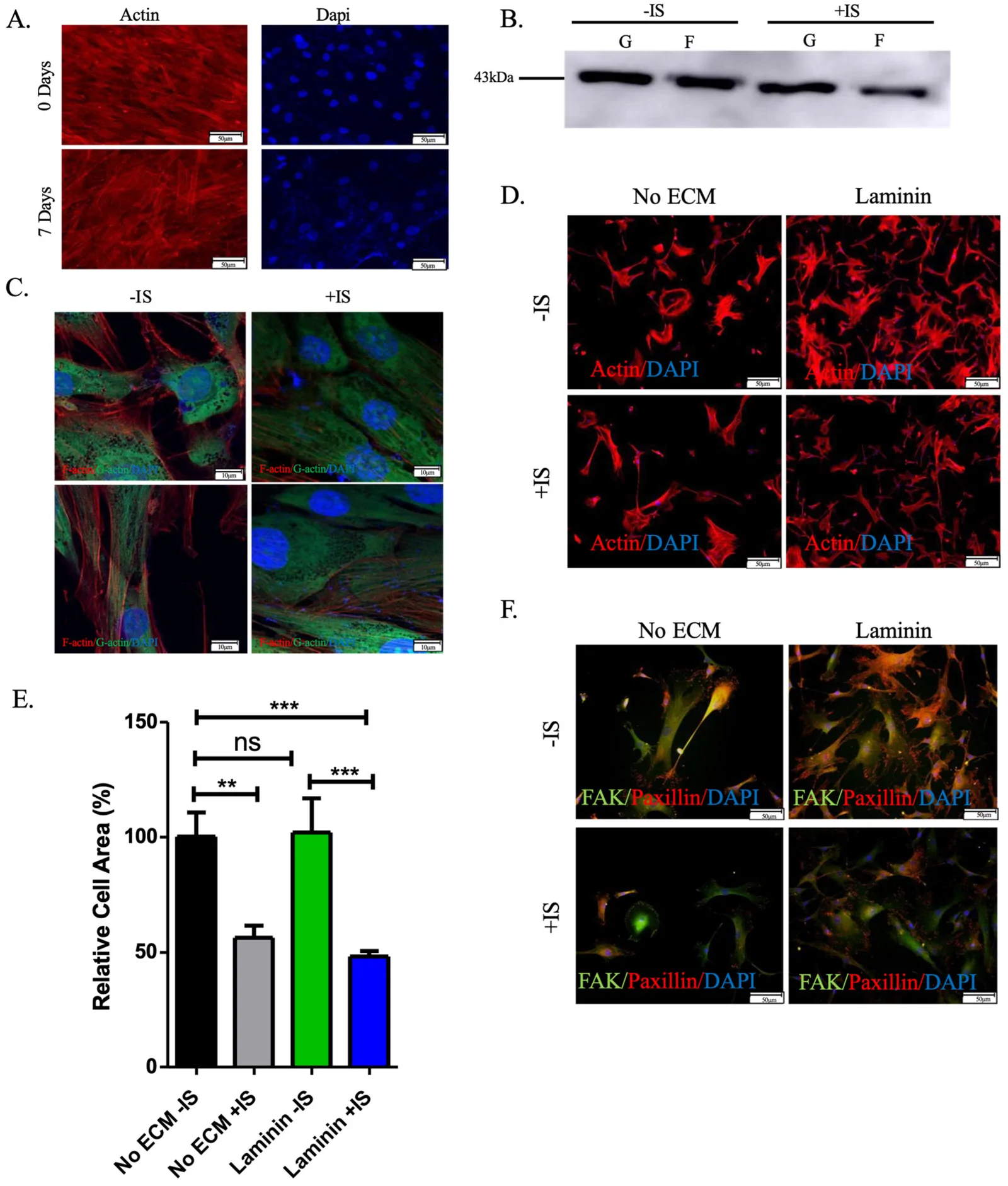
Indoxyl sulfate effect on actin cytoskeleton, cell size, and attachment. (**A**) Prolonged exposure to IS results in disorganization of the VSMC actin (actin = red, Dapi = blue) cytoskeleton (n = 3). (**B**) IS also results in decrease actin polymerization thus inhibiting actin fiber formation. There was decreased F-actin polymerization in IS exposed VSMCs. (n = 3). (**C**) Immunofluorescence staining of VSMCs for F- (Red) and G-Actin (Green). IS exposed cells have decreased F-Actin and increased G-actin pool which resulted in loss of actin fibers. (**D**) VSMCs were plated on standard-coating or laminin (20 µg/mL)-coated plates. This was to examine cellular size after IS exposure (n = 3). (**E**) Percentage of cell area with or without IS exposure. Demonstrating IS exposure resulted in decreased VSMC size (n = 3). (**F**) Focal adhesion in response to IS exposure. Focal adhesions decreased on standard-coating, and laminin-coated plates due to IS exposure (n = 3). ** *p* < 0.005, *** *p* < 0.0005.

IS-exposure induced actin cytoskeletal disorganization as evidenced through actin staining (Figure 3A). Within all cells, actin exists in two states, either filamentous-actin (F-actin) or globular-actin (G-actin), and the isolation of actin states demonstrated IS inhibited polymerization which will decrease the number of actin fibers (Figure 3B) [19,20]. Actin staining further reinforced the observation that IS-exposure inhibited actin polymerization and decreased the levels of F-actin (Figure 3C), lending further evidence that IS-exposure alters the VSMC actin cytoskeleton. The inhibition of actin polymerization will result in alterations to the morphology and directly drive the loss of motility previously observed.

IS-exposure significantly reduced the cell size by as much as 50% on laminin or standard ECM plates (Figure 3D,E) [35]. Alterations to the actin cytoskeleton led to smaller cells due to the lack of structural support to maintain larger cell sizes. A further analysis of VSMC ECM affinity was undertaken utilizing collagen and fibronectin coated slides. It was determined that fibronectin coating resulted in a decreased cell size in treated and untreated VSMCs. This is due to the role fibronectin has on VSMC phenotypic switching and effect on vascular injuries that leads to neointimal formation [36].

We found that VSMCs have an increased affinity for collagen and cell size (Supplementary Figure S3A–C). Gelatin increasing VSMC size is directly tied to the promotion of phenotypic maintenance and preserves cellular function [37]. VSMCs exposed to IS also experienced a change the integrin profile that affected attachment and response to the extracellular environment (Supplementary Figure S3D,E). Finally, to examine focal adhesions, which are attachment points for actin fibers, we utilized immunofluorescence staining for FAK and paxillin (Figure 3F) [38]. Focal adhesions are decreased after IS exposure leading to decreased actin attachment, and size. Taken together, this data demonstrated that IS-exposure directly interferes in the actin cytoskeleton structure, directly leading to alterations in cell size and attachment ability.

### 3.5. Focal Adhesion Loss Due to IS Exposure

**Figure 4:**
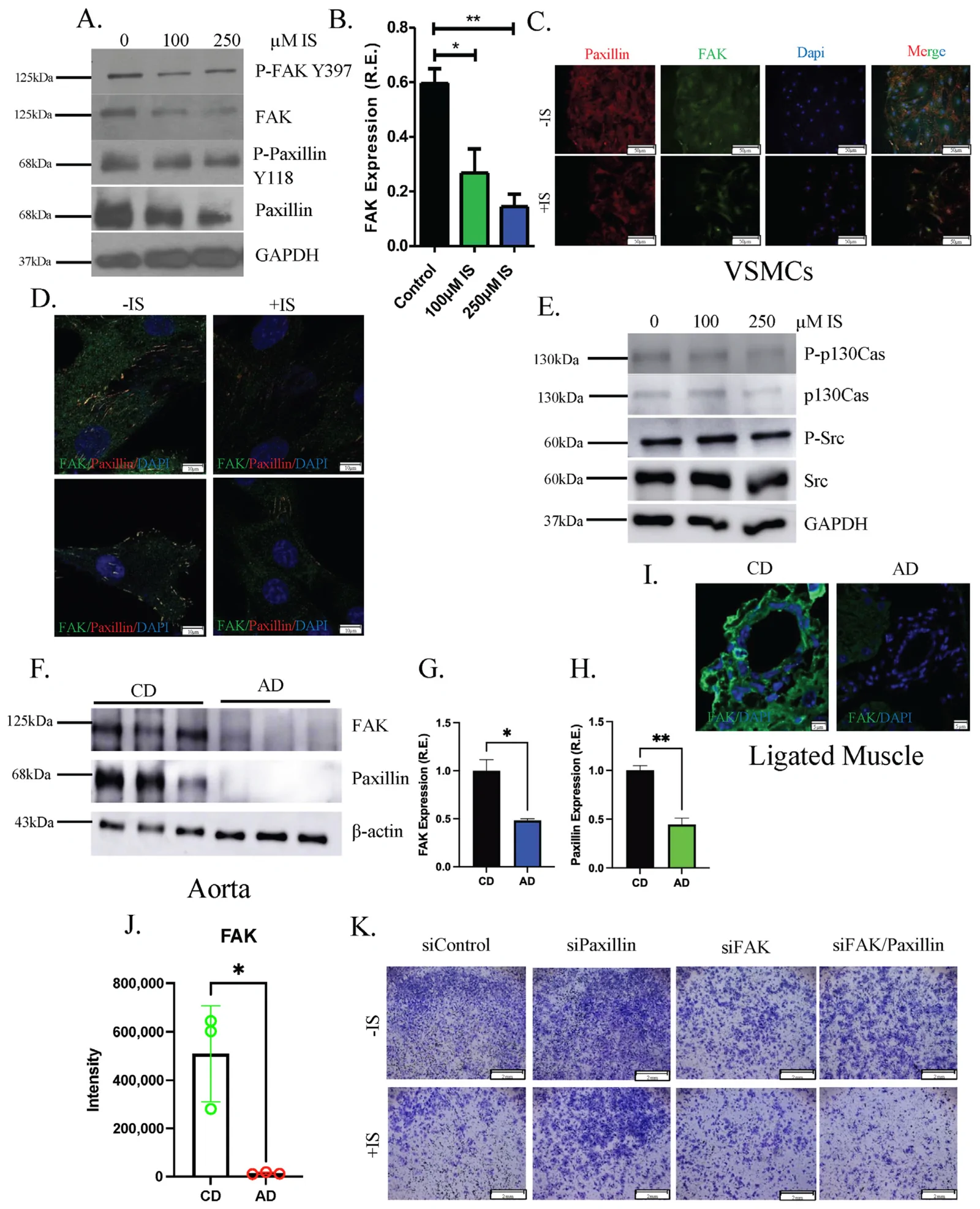
Indoxyl sulfate effect focal adhesions expression. (**A**) FAK and Paxillin decreased as IS concentration increases. Also results in loss of phosphorylation of p-FAK Y397 and p-Paxillin Y118. (**B**) FAK relative expression after IS-exposure (n = 3). (**C**) Focal adhesions made of FAK and Paxillin are decreased after IS-exposure at the leading edge. (**D**) Confocal microscopy further demonstrates the loss of focal adhesions. (**E**) Related focal adhesion protein p130Cas and Src are not affected by IS exposure (n = 3). (**F**) AD mice aortas were examined days post-surgery. FAK and Paxillin is decreased mimicking in vitro VSMC profile (n = 3). (**G**) Quantification of FAK demonstrates a significant decrease in AD mice aorta. (**H**) Quantification of Paxillin is significantly decreased in AD mice aorta (**I**) Ligated muscle from AD mice demonstrated lower expression of FAK in vessels (n = 3). (**J**) Quantification of FAK within ligated muscle was significantly decreased in AD mice (n = 3). (**K**) Silencing of either FAK, Paxillin, or both was examined via transwell migration. Silencing FAK had a marked decrease in migration however, this was not the case with Paxillin silencing (n = 3). (R.E.—Relative Expression) * *p* < 0.05, ** *p* < 0.005.

Dose-dependent IS-exposure resulted in a significant decrease in the expression of FAK and paxillin, and the activation of FAK at Tyr397 and paxillin at Tyr118 (Figure 4A,B) [39]. Microscopy demonstrated that FAK and paxillin were depleted at the leading edge of VSMCs and throughout the cell (Figure 4C,D). FAK and paxillin are vital components of focal adhesions; however, they are not the only proteins present, thus leading to the question of whether IS-induced loss is confined to these proteins. Another two canonical focal adhesion proteins Src and p130cas were examined for the activation and expression after IS-exposure. Analysis determined there was no alteration to activation or expression of these proteins due to IS-exposure (Figure 4E). An in vivo analysis of aortic samples recapitulated the in vitro samples, demonstrating a significant loss of FAK and paxillin in AD conditions (Figure 4F). An in-depth examination of vessels from ligated muscle in AD mice further demonstrated the loss of FAK (Figure 4G,H). These data demonstrate that CKD results in the loss of focal adhesion protein FAK due to the accumulation of IS. Without these points at the leading edge, there will be reduced actin polymerization and impaired forward motility.

It was also found that vessels lacked FAK expression after CKD induction. Ligated muscle was also examined for FAK expression. Compared to their counterparts in CD mice, ligated muscle from AD mice demonstrated a lower expression of FAK in vessels (Figure 4I). FAK or Paxillin were silenced in VSMCs, and the effect on migration was examined via transwell migration assay. The silencing of FAK resulted in significantly decreased VSMC motility; however, the loss of Paxillin had no effect on VSMC motility (Figure 4K). Consistent with those findings, in cultured VSMC, IS-exposed VSMCs exhibit decreased motility and loss of focal adhesion proteins.

It was necessary to elucidate if the loss of focal adhesions directly contributed to lost motility. We determined that the overexpression of FAK resulted in the restoration of IS-exposed VSMC motility when compared to IS-exposed VSMC (Supplementary Figures S3C and S4A). Taken together, these data demonstrate the loss of focal adhesion protein; specifically, FAK impairs vascular motility and plays a role in alterations in the actin cytoskeleton.

An analysis of VSMCs demonstrated that hypoxia did not alter the expression profile of FAK and Paxillin with or without IS exposure (Supplementary Figure S5A). Utilizing inhibitors for proteasome, and early- and late-stage lysosome degradation, we found that IS induced FAK and paxillin degradation through the proteasome degradation system (Supplementary Figure S5B).

**Figure 5:**
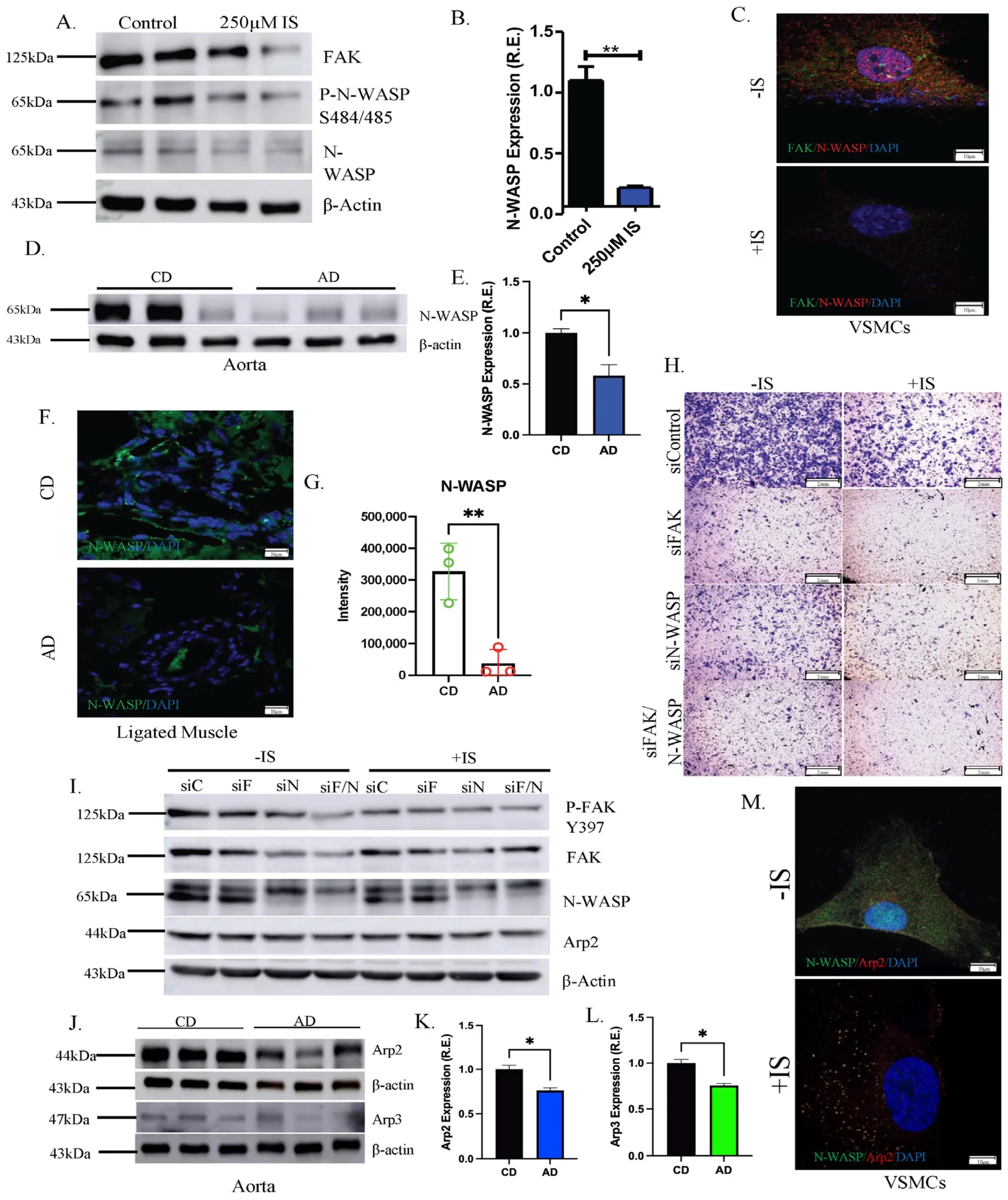
Downstream effect of indoxyl sulfate on FAK pathway. (**A**) N-WASP, a downstream effector FAK, has decreased expression and activation after IS-exposure in vitro in VSMCs. (**B**) N-WASP statistically decreased expression (n = 3). (**C**) FAK and N-WASP colocalization is decreased due to IS exposure. (**D**) Examination of N-WASP expression in aortic tissue from AD mice (n = 3). (**E**) N-WASP expression was decreased in vessels of ligated muscle from AD mice 28 days post-surgery (n = 3). (**F**) Quantification of N-WASP in mouse aorta demonstrated a significant decrease in AD. (**G**) Quantification of N-WASP within ligated muscle was significantly decreased in AD mice (n = 3). (**H**) FAK and N-WASP loss directly contribute to loss of VSMC migration after IS exposure (n = 3). (**I**) Silencing of FAK, N-WASP, or both were utilized to examine the effect on Arp2. Arp2 decreased with N-WASP silencing which results in decreased actin polymerization. (**J**) Arp2 and Arp3 decreased expression in AD mice (n = 3). (**K**) Quantification of Arp2 showing statistically down regulation in AD mouse tissue. (**L**) Quantification of Arp3 showing statistically down regulation in AD mouse tissue. (**M**) N-WASP and Arp2 colocalization also decreases after IS exposure demonstrating a loss of actin polymerization activity (n = 3). * *p* < 0.05, ** *p* < 0.005.

IS-exposure resulted in the significantly decreased expression of N-WASP and phosphorylation Ser484/485 (Figure 5A,B) [39,40,41]. Microscopy also demonstrated that IS-exposure altered the cellular localization of N-WASP, and decreased colocalization with FAK (Figure 5C). A further analysis of in vivo samples demonstrated that the AD diet led to a decreased expression of N-WASP (Figure 5D,E). Vessels from ligated muscle of AD-diet mice also exhibited a loss of N-WASP (Figure 5F,G). The loss of N-WASP directly contributed to an impaired VSMC motility in the same manner that the loss of FAK does (Figure 5H); IS-exposure and silencing of FAK and N-WASP caused the loss of Arp2, a major component of the Arp2/3 complex (Figure 5H), further demonstrating that IS-exposure directly stymied VSMC motility through the loss of FAK and N-WASP, and impaired actin polymerization [42,43].

Aortic tissue was used to assess whether CKD-associated IS accumulation alters FAK, N-WASP, and Arp2/3 expression in the systemic vasculature. To this end, the silencing of FAK, N-WASP, or both were utilized to examine the effect on Arp2. Arp2 decreased with N-WASP silencing, which results in decreased actin polymerization (Figure 5I).

Once it was understood that IS affected the FAK-N-WASP pathway which was involved in actin polymerization, the next step was to examine the Arp2/3 complex responsible for actin polymerization. An examination of aortic tissue from AD mice exhibited a clear loss of Arp2 and Arp3 (Figure 5J). The expression of Arp2 and Arp3 was directly downregulated in vivo models. N-WASP and Arp2 colocalization was altered and decreased after IS-exposure (Figure 5K,L). It was observed that N-WASP and Arp2 localization was restricted in a punctate distribution pattern (Figure 5M) and N-WASP and Arp2 colocalization also decreases after IS exposure (Figure 5M). Taken together, it has been demonstrated that aberrations to actin polymerization are responsible for lost motility. The loss of actin polymerization and control pathways would directly impair vascular regeneration, microvascular remodeling, and vessel maturation.

## 4. Discussion

Vascular complications are a well-established hallmark of CKD progression [44]. This study demonstrated that uremic toxin IS directly impaired vascular regeneration and microvascular remodeling through alterations to the actin cytoskeleton, disruptions to actin polymerization, loss of focal adhesions, and cellular attachment. IS-exposure drastically altered the expression of canonical focal adhesion proteins FAK, and Paxillin in both in vitro and in vivo models. These major components of focal adhesions are necessary for proper formation and structure. The loss of focal adhesions, due to uremic toxin accumulation, will interfere in response to extracellular stimuli necessary for vascular regeneration. Impaired vascular regeneration due to the loss focal adhesions would account for data from CKD mice which exhibited defective blood flow restoration. Understanding how focal adhesion are involved in vascular regeneration during CKD progression is necessary.

Vascular regeneration and microvascular maturation are essential processes responsible for functional blood vessels following ischemic injury. Impaired vascular repair can contribute to tissue ischemia, necrosis, ulceration, infection, lower-extremity amputation, and, ultimately, patient mortality [45]. Defective microvascular remodeling can lead to tissue and delayed wound healing. Functional microvascular recovery requires endothelial cell proliferation and vessel formation, followed by the recruitment of VSMCs, to support vessel stabilization and maturation. VSMCs are particularly important for the structural remodeling of small muscular vessels and arterioles, providing mechanical support and regulating vascular tone. Inadequate VSMC coverage can compromise vascular stability and limit effective tissue perfusion. Consequently, impaired microvascular maturation will contribute to prolonged recovery and increased risk of ischemic complications in CKD patients [45,46]. Understanding how CKD and uremic toxin accumulation disrupt these processes is therefore essential for identifying mechanisms underlying defective vascular regeneration.

Particular attention was paid to FAK, which regulates cytoskeletal organization, cellular adhesion, motility, and the transmission of extracellular signals. Our findings demonstrate that IS-exposure significantly reduced FAK expression and impaired VSMC motility. Because VSMC migration contributes to vessel wall remodeling and maturation, disruption of FAK signaling may compromise the structural adaptations of the microvasculature during ischemic injury. These findings support and association between CKD-related FAK dysfunction and impaired vascular regeneration. To further investigate the molecular mechanisms underlying reduced motility, we examined downstream regulators of actin polymerization. Our results demonstrated that N-WASP expression was significantly decreased following IS-exposure and during CKD progression. N-WASP belongs to the Wiskott–Aldrich Syndrome (WAS) protein family which regulates actin cytoskeletal dynamics. N-WASP activates the Arp2/3 complex, a major regulator of branched actin polymerization required for membrane protrusions and cellular migration. Alterations to Arp2/3 activation or expression, as demonstrated in our analysis, would directly inhibit actin fiber formation [47,48]. Our analysis further demonstrated that decreased Arp2/3 expression following IS-exposure, consistent with the observed disruption of actin organization and altered VSMC morphology. Together, these findings demonstrate that IS disrupts the FAK-N-WASP-Arp2/3 signaling axis, impairing the cytoskeletal organization and migratory capacity required for effective microvascular remodeling. These alterations contribute to reduced VSMC coverage of developing or remodeling vessels, impaired vascular maturation, and inadequate perfusion recovery following ischemic injury.

Shimizu et al. demonstrated conflicting results regarding the effects of IS-exposure on VSMC motility. They showed that IS-exposure could increase VSMC migration through the activation of PDGFRβ, a signaling pathway involved in VSMC recruitment and microvascular remodeling. However, there are fundamental differences between our study and their experimental approach. They did not examine prolonged IS-exposure in the absence of sustained PDGF-ββ stimulation. Additionally, IS was employed primarily as a chemotactic agent rather than as a prolonged toxicant capable of inducing cellular changes. Our work demonstrates the effects of prolonged IS-exposure on VSMC migration and function, which are important cellular processes involved in microvascular remodeling and maturation [49].

The demonstration of the adverse effects CKD progression and IS accumulation has on vascular regeneration, microvascular remodeling, and maturation provides insights into previous clinical reports of vascular damage [44]. CKD patients experience multiple vascular complications that can be associated with impaired vascular regeneration and defective microvascular remodeling. Firstly, CKD patients presenting with abdominal aortic aneurysms who have undergone surgical repair are at increased risk mortality due to complications, including repair failure. Secondly, vascular canulation sites used for hemodialysis in CKD patients are vulnerable to vessel leakage and impaired vascular integrity [8]. Finally, CKD and diabetic patients who undergo extremity amputation exhibit decreased would healing and elevated rates of mortality [9]. Impaired microvascular remodeling and maturation can contribute to these complications by limiting vessel stability, VSMC coverage, and effective tissue perfusion. Our data could provide insights into the molecular basis of these vascular abnormalities, particularly through the loss of FAK signaling and impaired actin polymerization, which disrupt VSMC migration and cellular processes necessary for microvascular remodeling and maturation. These findings provide a foundation for developing potential mitigation strategies and therapeutic interventions to improve vascular repair in CKD patients.

By demonstrating that uremic toxins directly impact vascular health, microvascular remodeling, and repair capacity it becomes necessary to investigate future mitigation strategies. In this study, we describe vascular dysfunction that has been partially observed in clinical settings but has not been fully examined at the mechanistic, or phenotypic level. Renal specialists have identified numerous complications arising from vascular injuries, but the relationship between CKD and defective microvascular repair mechanisms remains incompletely understood. By understanding how IS accumulation contributes to impaired microvascular remodeling and maturation, we can better direct therapeutic strategies to improve vascular recovery and potentially reduce mortality in CKD patients. These approaches can include improved dialysis methods to remove circulating uremic toxins or direct inhibition of hepatic IS production by targeting sulfotransferase Sult1A1 [50,51,52]. By understanding the role IS plays in vascular injury and defective microvascular maturation, these findings can open new therapeutic avenues to treat or potentially prevent vascular complications. Along with inhibiting IS formation by sulfotransferases, it is possible to prevent precursor formation via dietary tryptophan restriction. The inhibition of IS-producing pathways can decrease circulating IS levels and mitigate vascular damage. Another approach to reducing serum IS concentrations involves preventing the absorption of indole, a metabolite derived from bacterial tryptophan metabolism. This can be achieved through the oral sorbent AST-120, which binds indole and related compounds in the intestinal tract, limiting absorption and promoting excretion [53]. However, tryptophan restriction as a treatment strategy remains controversial. Studies have demonstrated both beneficial and adverse consequences associated with restriction [54,55]. Tryptophan is an essential amino acid required for the production of multiple biologically relevant metabolites. Insufficient tryptophan availability can alter metabolite production and negatively affect neurological function, mental health, and sleep. Understanding how to regulate tryptophan metabolism while preserving its essential physiological function is therefore important for developing effective treatments. Additional studies are necessary to elucidate the contributions of individual tryptophan metabolites to CKD pathology and impaired microvascular remodeling.

Our findings identify the FAK–N-WASP–Arp2/3 signaling axis as a potentially important mechanistic link between uremic toxin accumulation and impaired vascular regeneration and microvascular remodeling in CKD. Because VSMC migration and recruitment are essential for microvascular maturation, vessel stabilization, and structural remodeling, the disruption of FAK-dependent actin dynamics by IS may contribute to the reduced VSMC coverage and impaired microvascular remodeling observed in CKD. These findings also highlight potential therapeutic opportunities aimed at preserving vascular function in patients with CKD. Strategies that reduce circulating IS levels, including approaches such as AST-120, may limit the detrimental effects of uremic toxins on VSMCs, while interventions that preserve or restore FAK signaling could enhance N-WASP–Arp2/3-dependent actin remodeling and VSMC motility. Although additional studies are required to establish a direct causal relationship between restoration of this pathway and improved microvascular remodeling and maturation in vivo, targeting IS accumulation and the FAK–N-WASP–Arp2/3 axis represents a promising direction for future investigation. Ultimately, preserving VSMC migratory capacity and microvascular remodeling may improve tissue perfusion and reduce the burden of ischemic vascular complications in patients with CKD.

When examining microvascular remodeling in several limitations should be considered. Currently, there are shortcomings in fully examining microvascular remodeling and maturation because sufficient models have not been established to directly replicate these complex processes. In vitro models are limited in their ability to reproduce microvascular remodeling due to the absence of appropriate extracellular matrix and the cellular interactions necessary for vessel maturation. Microvascular remodeling is a complex process that involves the formation of capillary networks by endothelial cells, followed by the recruitment of VSMCs to provide structural support and promote vessel stabilization. However, many questions remain concerning the exact cellular interactions and environmental factors necessary to achieve proper microvascular maturation, vessel stability, and blow flow capacity. We are also limited in our understanding of how CKD mitigation strategies including, hemodialysis and oral sorbents to reduce intestinal absorption of uremic toxins, will influence observations in this study. Another limitation of the current study is the absence of a FAK rescue experiment to directly establish whether restoration of FAK signaling is sufficient to reverse IS-induced impairments in VSMC motility. Future studies incorporating FAK rescue or reactivation are warranted to establish causality and further define the therapeutic potential of targeting the FAK–N-WASP–Arp2/3 signaling axis.

## 5. Conclusions

Our study provides compelling evidence that uremic toxins, both in vitro and in vivo, have deleterious effects on vascular regeneration and microvascular remodeling. By elucidating the molecular mechanisms altered by IS-exposure, particularly, disruptions in FAK-dependent actin polymerization and VSMC migration, our study provides novel insights into impaired microvascular maturation during CKD progression and opens new avenues for potential therapeutic strategies aimed at improving vascular regeneration.

## Data Availability

The data will be made available upon reasonable request.

## Supplementary Materials

The following supporting information can be downloaded at https://www.mdpi.com/article/10.3390/cells15211946/s1.

## Abbreviation

Abbreviation	Definition
FAK	Focal Adhesion Kinase
Nrp1	Neuropilin 1
N-WASP	Neuronal Wiskott–Aldrich Syndrome Protein
IS	Indoxyl Sulfate
PWV	Pulse Wave Velocity
EF	Ejection Fraction
FS	Fraction Shortening
CKD	Chronic Kidney Disease
VSMC	Vascular Smooth Muscle Cells
T2D	Type II Diabetes
IRS1	Insulin Receptor Substrate 1
AAA	Abdominal Aortic Aneurysm
PAD	Peripheral Artery Disease
ECM	Extracellular Matrix
