# Supplementary material for: Inhibition of FAK-Actin Pathway by Indoxyl Sulfate Suppresses Microvascular Remodeling in a Mouse Model of Chronic Kidney Disease
Source: Cells. 2026 Oct 26;15(21):1946. doi: 10.3390/cells15211946 (PMC13762078; doi:10.3390/cells15211946)
Supplement: Supplementary file 1 [file cells-15-01946-s001.zip › cells-4491381-supplementary.pdf]

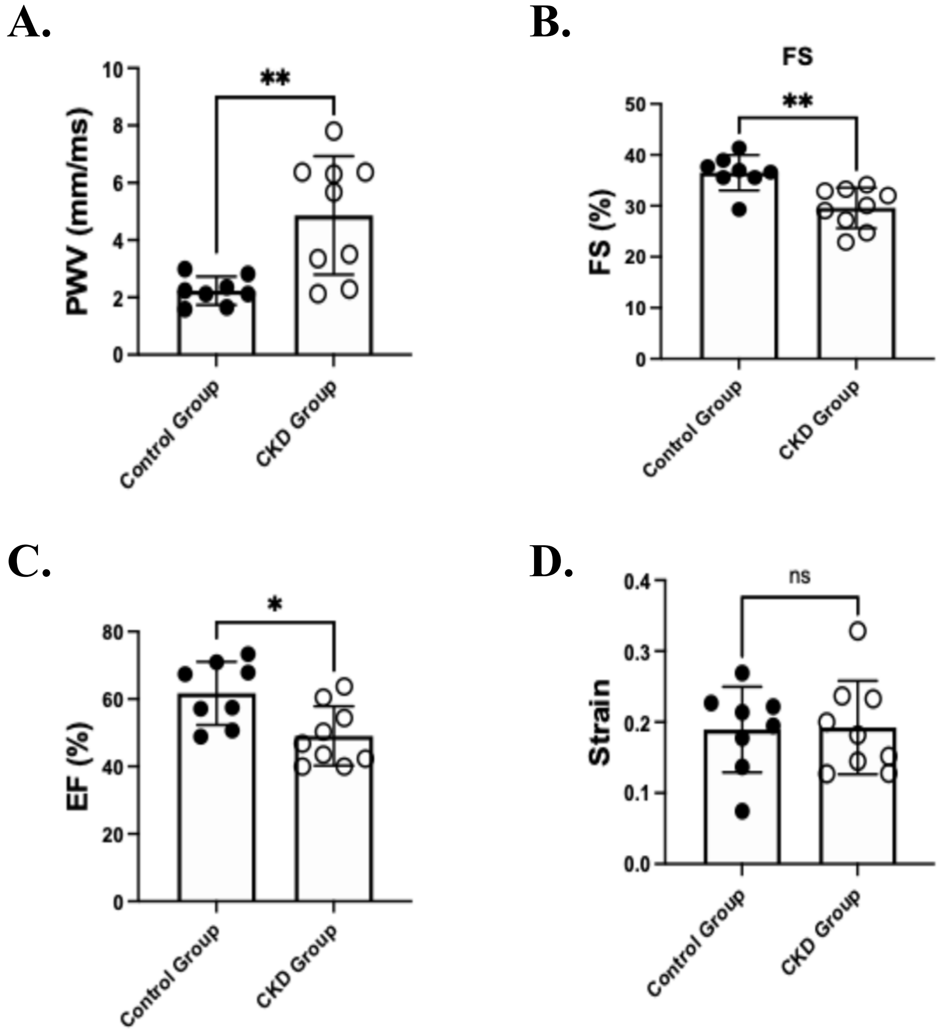

Supplementary Figure 1: Cardiovascular Complications in the CKD mice. A. Pulse Wave Velocity (PWV) was increased due to CKD. B. Fraction Shortening (FS) was elevated in CKD conditions. C. Ejection Fraction (EF) was decreased in CKD conditions. D. Vascular strain was not significantly affected by CKD conditions. (Control Group n=8, CKD Group n=9)

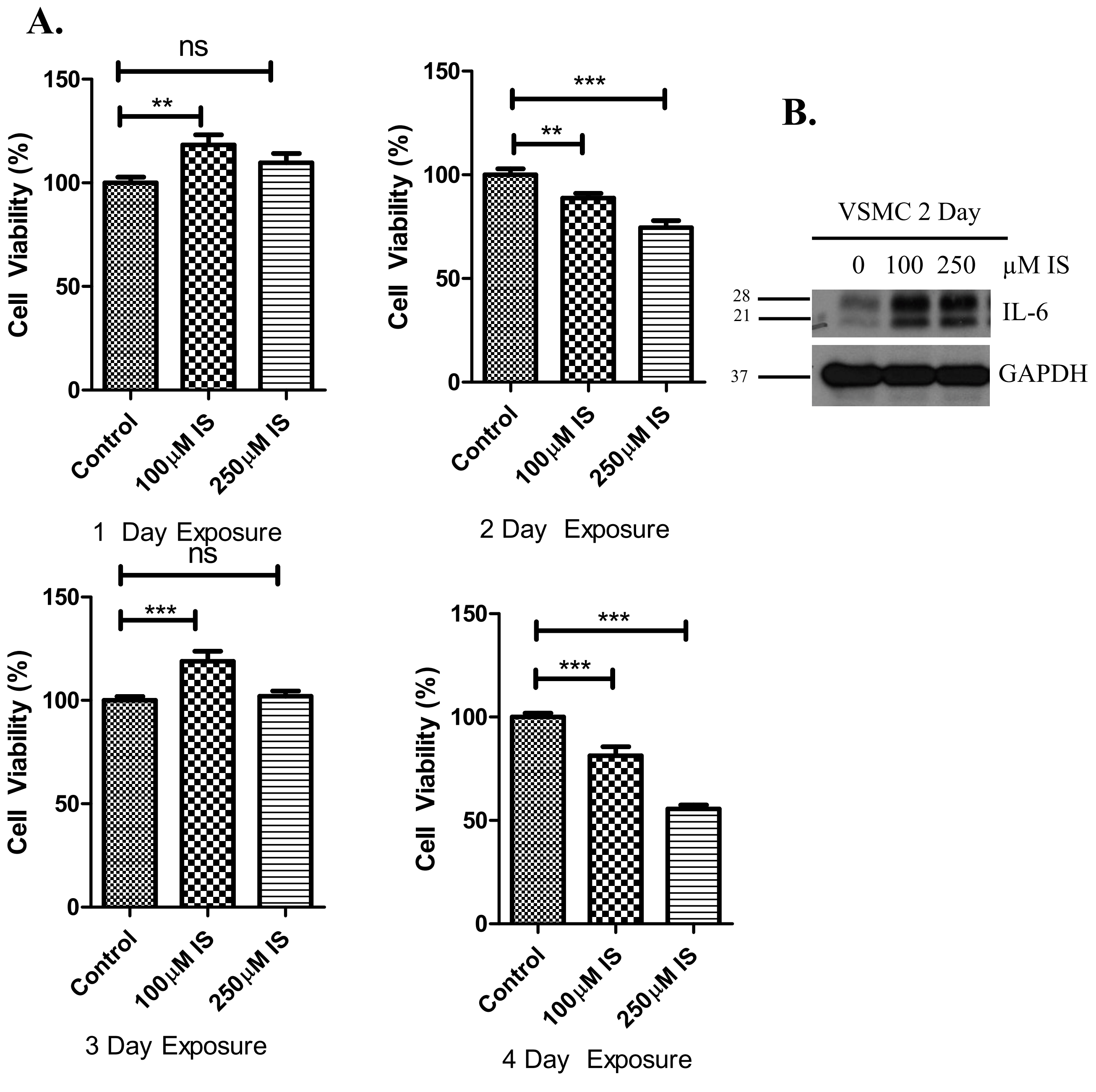

Supplementary Figure 2: **A**. VSMC viability after IS-exposure from 1 to 4 days. **B**. VSMCs experience inflammation due to IS but not cell death. **$p<0.005$, ***$p<0.0005$

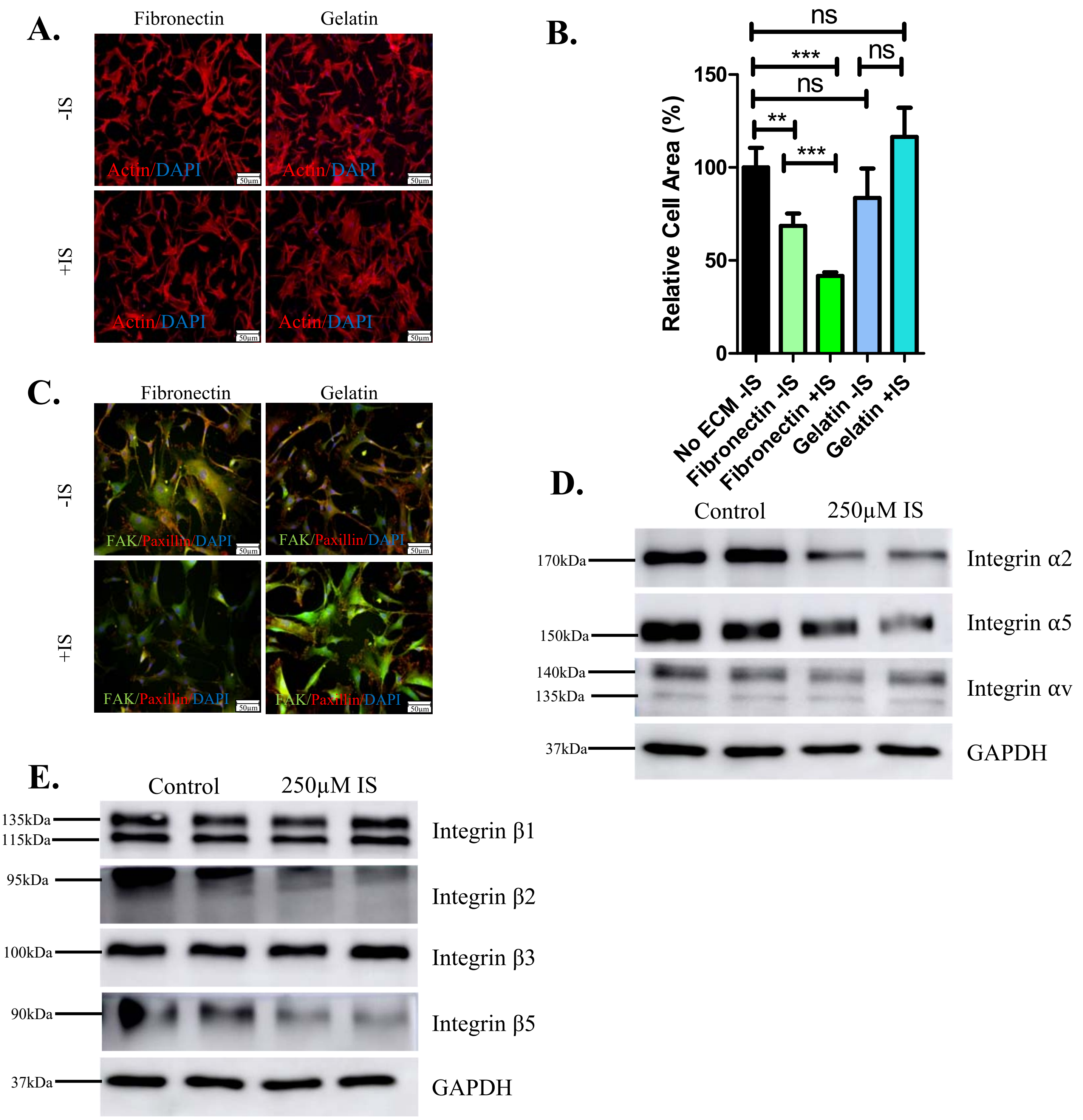

Supplemental Figure 3: IS-exposure leads to alterations of cell size and attachment ability. A. Fibronectin (10ng/mL) and Gelatin (Cellbiologic). Cell size decreased on Fibronectin after IS exposure and increased on Gelatin. **B.** Graph of cell size for fibronectin and gelatin. C. Focal adhesions change on Fibronectin and Gelatin. D&E. Integrins profile for both alpha and beta subunitss shifted in VSMCs after IS exposure (n=3). **p<0.01, ***p<0.005

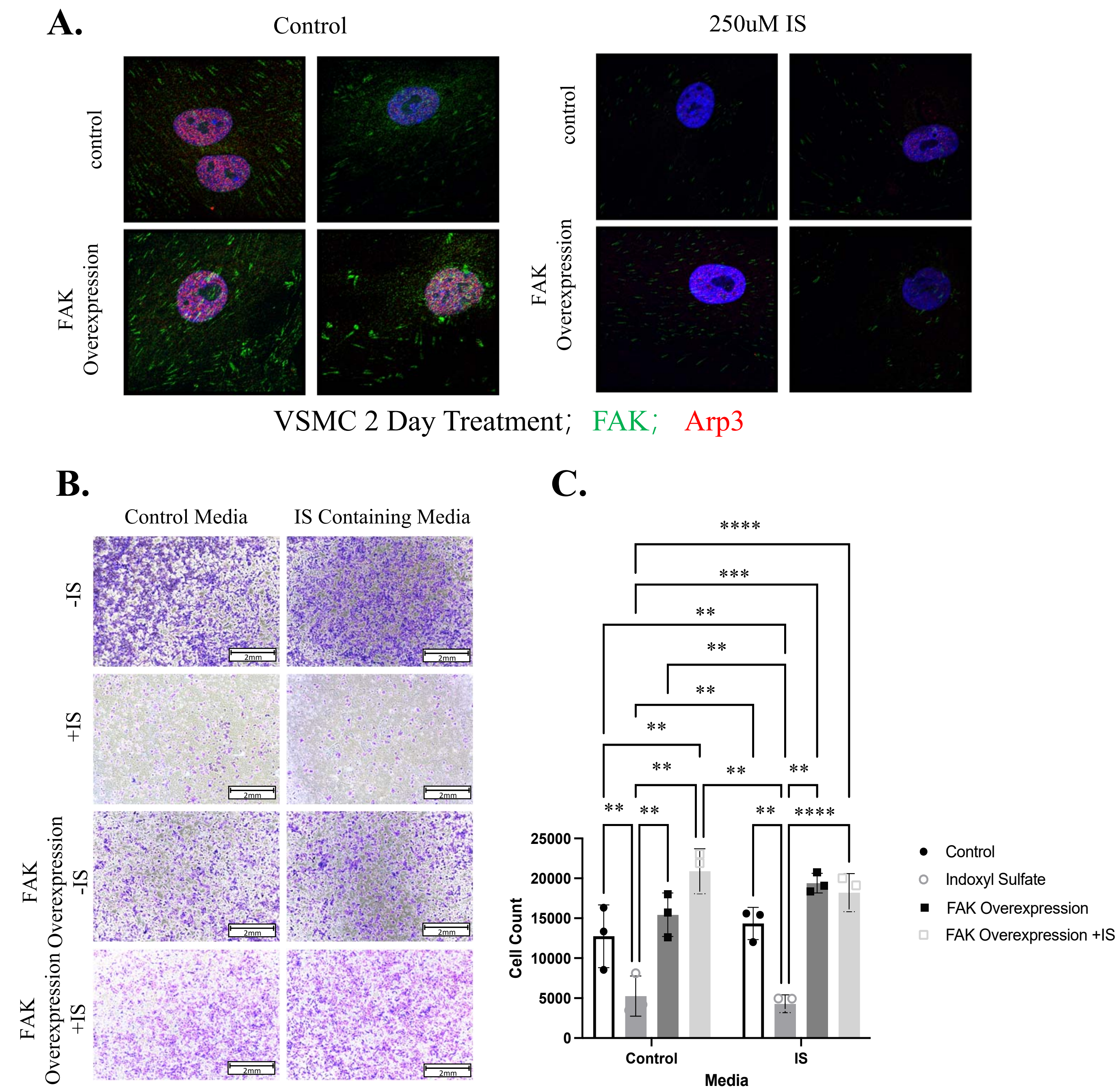

Supplementary Figure 4: A. Overexpression of FAK was employed in VSMCs. **B**. VSMCs overexpression of FAK in IS-exposed VSMCs there was restored motility compared to IS-exposed VSMCs (n=3). **C**. Statistical analysis of cell counts (n=3). **$p<0.01$, ***$p<0.005$ ****$p<0.0001$

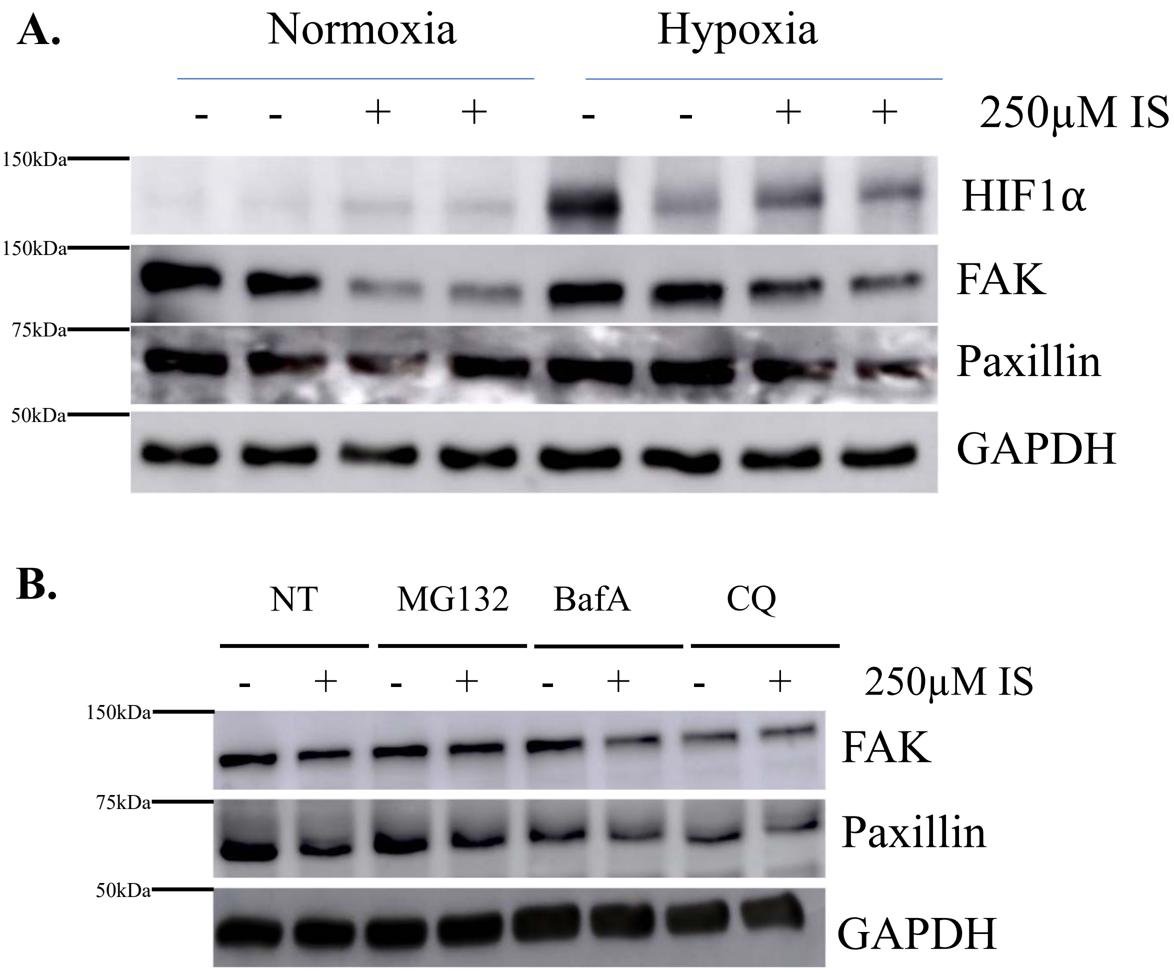

Supplementary Figure 5: Effects of hypoxia and MG132 on IS-induced FAK and Paxillin loss in vitro. **A**. Examination of the effect of 12hrs Hypoxia on VSMCs exposed to IS. Hypoxia was unable to rescue FAK and Paxillin expression (n=3). **B**. Inhibition of protein degradation after IS exposure indicating that IS induces FAK and Paxillin loss through proteasome degradation (n=3).
